# Interactions between Natural Health Products and Oral Anticoagulants: Spontaneous Reports in the Italian Surveillance System of Natural Health Products

**DOI:** 10.1155/2011/612150

**Published:** 2011-01-09

**Authors:** Angelica Paoletti, Eugenia Gallo, Silvia Benemei, Michele Vietri, Francesco Lapi, Roberta Volpi, Francesca Menniti-Ippolito, Luigi Gori, Alessandro Mugelli, Fabio Firenzuoli, Alfredo Vannacci

**Affiliations:** ^1^Preclinical and Clinical Pharmacology Department, Tuscan Regional Centre of Pharmacovigilance, University of Florence, Viale Pieraccini 6, 50139 Florence, Italy; ^2^Clinical Chemistry Laboratory, Spedali Civili Hospital, Brescia, Italy; ^3^National Centre of Epidemiology, Surveillance and Health Promotion, National Institute of Health, Rome, Italy; ^4^Centre of Natural Medicine, S. Giuseppe Hospital, Empoli, Italy

## Abstract

*Introduction*. The safety of vitamin K antagonists (VKA) use can be compromised by many popular herbal supplements taken by individuals. The literature reports that 30% of warfarin-treated patients self-medicates with herbs. Possible interactions represent an health risk. We aimed to identify all herbs-oral anticoagulants interactions collected in the Italian database of suspected adverse reactions to “natural health” products. *Methods*. The Italian database of spontaneous reports of suspected adverse reactions to natural products was analyzed to address herb-VKAs interactions. *Results*. From 2002 to 2009, we identified 12 reports with 7 cases of INR reduction in patients treated with warfarin (*n* = 3) and acenocoumarol (*n* = 4), and 5 cases of INR increase (all warfarin associated). It was reported 8 different herbal products as possibly interacting. *Discussion*. Our study confirms the risk of interactions, highlighting the difficulty to characterize them and their mechanisms and, finally, prevent their onset. The reported data underline the urgent need of healthcare providers being aware of the possible interaction between natural products and VKA, also because of the critical clinical conditions affecting patients. This is the first step to have the best approach to understand possible INR alterations linked to herb-VKA interaction and to rightly educate patients in treatment with VKA.

## 1. Introduction

Warfarin and other vitamin k antagonists (VKAs) are widely used as oral anticoagulants, for treatment or prevention of thromboembolism, in particular, in case of atrial fibrillation. Oral anticoagulants have a narrow therapeutic index; therefore, even small changes in plasma concentration could lead to marked alterations in therapeutic effect and/or toxicity. Despite the possibility of monitoring the anticoagulation status by measuring prothrombin time (PT) expressed as international normalised ratio (INR), the management of therapy is challenging because those parameters usually show unpredictable inter- and intraindividual variability. Gender, age, ethnicity, and vitamin K intake (foods and herbs) can contribute to this instability, by pharmacokinetic and pharmacodynamic interactions. Genetic polymorphisms in vitamin K metabolizing enzymes also correlate with dose requirements. Case reports and case series represent the bulk of evidence regarding herb-warfarin interactions [1, 2], even if most reports do not deal with mechanisms, being “theoretical speculations” the sole bases for the analysis of potential interactions. Despite these concerns, several surveys reported that about 30% of warfarin-treated patients self-medicate with herbal remedies [1]. Use of herbal drugs and nonconventional medicine in Italy is quite diffuse, with almost 9 million people (13.6% of the Italian population) using at least one unconventional therapy during the year 2005; herbal medicine in particular is used by 3.7% of Italian population [3]. 

In this frame, since the Italian Pharmacovigilance System (Italian Medicines Agency—AIFA) only collects reports for registered drugs, the Italian National Institute of Health (Istituto Superiore di Sanità—ISS) sets up a specific adverse reaction reporting system for natural health products, by means of which from April 2002 to December 2009, 379 spontaneous reports of suspected adverse reactions to natural health products were collected. In the first phase of the surveillance, reports were sent mainly from few clinical centres in northern Italy, while the number of different reporters increased, including hospital doctors, general practitioners, and pharmacists from most Italian regions.

## 2. Methods

In the present study, the Italian database of spontaneous reports of suspected adverse reactions to natural products was analyzed to address herb-VKAs interactions. Patients, showing INR modifications after herbal drugs assumption, were included in the analysis only if they were previously stably anticoagulated (>65% of time in therapeutic range and at least 3 consecutive INR evaluation in therapeutic range before the reported case). 

The probability that the adverse events were the result of drug interaction was assessed in accordance to a modified Naranjo scale, the Drug Interaction Probability Scale specifically adapted for warfarin-dietary supplements interactions [1]. Factors interfering with VKAs (illnesses, dietary vitamin K intake changes) were excluded.

## 3. Results

From 2002 to 2009, we identified 12 reports with 7 cases of INR reduction in patients treated with warfarin (*n* = 3) and acenocoumarol (*n* = 4), and 5 cases of INR increase (all warfarin associated). 

### 3.1. Reduction of Anticoagulant Effect

Seven cases showed a significant reduction of INR (Table 1), discovered after routine coagulation blood test 4 days to 1 month after the start of herbal preparations consumption. Patients were in chronic therapy with warfarin (*n* = 3) or acenocoumarol (*n* = 4). Partial or complete normalization of INR was obtained after herbal preparations withdrawal (dechallenge) and increase in VKAs dosage. Six events were assessed as “possible” and one as “probable.” 

In one case, the INR reduction occurred with concomitant intake of warfarin and home-made aloe preparation. Although in the medical literature no evidence is reported of reduction of anticoagulant efficacy due to specific aloe-warfarin interactions, it is known that anthranoid plants (such aloe leafs) can influence the absorption of drugs by accelerating the gastrointestinal emptying, thereby decreasing their bioavailability. It has been reported that aloe exerts antiplatelet effects, due to its salicylates content: a case report describes a massive bleeding due to the interaction between aloe and sevoflurane, possibly due to the pharmacodynamic antiplatelet effects of these two agents [4].

We also reported the possible interaction between acenocoumarol and red ginseng. INR decline associated with coadministration of ginseng and warfarin has been reported [5]. The mechanism of ginseng-anticoagulant drugs interaction has not been elucidated, it could be that ginseng reduces the anticoagulant effect of warfarin through the induction of CYP450 liver enzymes. In a randomised, double blind, placebo controlled trial, after 2 weeks of ginseng administration, healthy subjects showed a statistically significant reduction of peak INR, warfarin peak plasma level and area under the curve in comparison with the placebo group [6]. However, the effect of ginseng on CYP450 is still controversial. 

A reduction of anticoagulant effect was observed in a patient taking a fermented preparation made from yeast fermentation of papaya and in a patient taking papaya extract. However, papaya assumption has been associated with the increase of warfarin effect through the inhibition of platelet aggregation [7]. The fermentated product has a high content in *β*-glucans, which may be involved in the reduction of the anticoagulant effect of warfarin since they could promote coagulation, interacting with its extrinsic pathway, probably by inducing monocyte tissue factor expression in human whole blood [8]. For the other case, we have not an explanation of the effect produced.

In one case, INR reduction occurred after assumption of warfarin and bilberry concentrate juice. We found no similar reports in the literature. The reduction of warfarin activity is unexpected since, the bilberry anthocyanosides, retaining antiplatelet properties, should increase the bleeding risk when co-administered with anticoagulant and antiplatelet drugs [9]. The interaction mechanism is unknown. Twelve cases of possible interaction between cranberry and warfarin have been reported to the Committee on Safety of Medicine in the UK, including eight cases with increased INR and/or bleeding episodes, three cases of unstable INR, and one case of decreased INR. INR increase in concomitance with cranberry juice intake has been described [10] with multiple possible mechanisms of action. These reports suggest that patients on warfarin therapy should be discouraged from drinking juice of plants belonging to the *Vaccinium Species*.

One case of INR reduction following the assumption of dietary supplement containing several vitamins (C, E, A) and fish oil was reported. According to the literature, its components could afford an increase of INR instead of a reduction. In fact, vitamin A in high doses was reported to prolong prothrombin time and induce bleeding in subjects treated with warfarin, although this evidence is based on few case reports and with un unclear mechanism of action [11]. The same is for vitamin E [11] and fish oil, reported to affect platelet aggregation and/or vitamin K-dependent coagulation factors [12]. Because of our case and the scarcity of evidence for INR-increase, further investigations should be conducted.

Finally, we report a case of INR reduction after oral intake of a dietary supplement containing green tea and other herbal extracts. Green tea contains vitamin K, which may directly antagonize the anticoagulant effect of coumarin drugs. Brewed green tea contains low amounts of vitamin K [13]; therefore, large quantities of brewed green tea would be necessary to have a significant changes in INR. However, the possible inhibitory effect of green tea on warfarin should be kept in mind.

### 3.2. Increase of Anticoagulant Effect

Many herbs, such as Dan Shen and Dang Gui, have been reported to enhance warfarin anticoagulation, as documented by the INR increase. Compounds like licorice, passionflower, and arnica contain coumarin derivates. Other compounds, including garlic and ginkgo, increase the bleeding risk associated with coumarin inhibiting platelet aggregation [1]. We report 5 cases of INR increase in warfarin-treated patients 10–30 days after consumption of arnica- or boswellia-based products (Table 2).

One patient showed the INR elevation after 10 days of assumption of arnica tablets for myalgia. Complete recovery was achieved after dechallenge. 

Another patient had a serious adverse event, with elevation of INR after 1 month of arnica homeopathic cream application for myalgia. After dechallenge and vitamin-K treatment, INR returned in therapeutic range. Causal relationship was assessed as “possible” for both events. In the second case reported, we hypothesize that systemic adsorption from the topic application site has occurred, allowing coumarin derivates contained in Arnica, as scopoletin and unbelliferone [11], to induce over-anticoagulation. 

In two other cases, INR increase occurred with concomitant intake of warfarin and *Boswellia serrata*. In both cases, complete recovery was achieved after dechallenge, and the causality relationship was defined as “probable.” To our knowledge, these are the first reports of interaction between warfarin and boswellia. The mechanism underlying this interaction is unknown, but it has been recently demonstrated that boswellic acids, assumed as the anti-inflammatory principles of the *Boswellia Species*, are able to inhibit lipoxygenase and directly interfere with COX-1 [14]. Cyclooxygenases inhibitor drugs (e.g., acetylsalicylic acid) interact with warfarin provoking an increased risk of bleeding, mainly as a result of platelet aggregation inhibition, although not accompanied by a change in INR. Furthermore, different species of boswellia (including *Boswellia serrata*) could inhibit CYP2C19, CYP3A4, and CYP 2C9 [15], the most important isoenzyme metabolizing S-warfarin, possibly increasing warfarin activity. It should be also considered that genetic variants of the CYP2C9 may contribute to an increase in the risk of overcoagulation due to interaction INR increase (up to 8) was reported also in a patient taking three multiherb products, including *Boswellia serrata*. Some of the assumed herbs contain coumarin derivates, but the high number of different plants made it impossible to identify the interacting components. However, multi-ingredient products should be strongly discouraged, especially in patients taking anticoagulants, due to the high risk of unknown toxicity and drug interactions by synergic effects.

## 4. Discussion and Conclusions

Our analysis confirmed the risk of interactions between herbs and VKAs, possibly exiting in adverse events [16]. Limited information about the pharmacokinetics, pharmacodynamics, and manufacturing properties of herbal and dietary supplements leads to difficulty in characterizing and predicting interactions and understanding their mechanisms. Although the use of herbal products may not be dangerous per se, lack of communication between patient and physician might alter clinical management of adverse events. Healthcare providers should be aware of potential interactions and their role in patients' education should be active. Physicians should be encouraged to discuss complementary/alternative therapies with their patients putting an end to the “Do not ask! Do not tell!” approach that characterized communication in this area. This is the first step to have the best approach to understand possible INR alterations linked to herb-VKA interaction and to rightly educate patients in treatment with VKA.

## Figures and Tables

**Table 1:** Reports of INR reduction associated with use of natural remedies.

Sex, age (years)	Product, dosage, and indication	Concomitant medications and notes	Adverse drug reaction, time to onset, and Naranjo score	Action taken and outcome
F, 45	*Camellia sinensis, Malva sylvestris, Elettaria cardamomum, Hibiscus sabdariffa * (THERMOJETICS, Herbalife International of America, Inc Los Angeles, CA), 3 g/day; weight loss diet	Acenocoumarol 14 mg/w (since 10 years)	Reduction of anticoagulant therapy efficacy INR 1.4; 16 days; Possible [4]	Herbal drugs withdrawn, Acenocoumarol dosage increase (16 mg/w); completely recovered

F, 70	*Aloe barbadensis, *honey, alcohol beverage, herbal liquid home-made preparation, 3 spoons/day; constipation	Warfarin 5 mg/w (since 10 years)	Reduction of anticoagulant therapy efficacy INR 1.42; 2 weeks; Possible [3]	Herbal drug withdrawn, warfarin dosage increase (6.25 mg/w); completely recovered

F, 81	*Panax Ginseng* (Red Ginseng), dosage not reported; tiredness and dizziness	Acenocoumarol 7 mg/w	Reduction of anticoagulant therapy efficacy INR 0.99; Possible [4]	Herbal drugs withdrawn, Acenocoumarol dosage increase (08 mg/w); completely recovered

M, 70	Fermented Papaya Preparation (IMMUN'AGE FPP, Osato international, Gifu, Japan), 3 g/day orally; ageing prevention	Warfarin 25 mg/w (since 9 years)	Reduction of anticoagulant therapy efficacy INR 1.64; 1 month; Probable [8]	Herbal drug withdrawn, warfarin dosage increase (27.5 mg/w); completely recovered

F, 80	*Vaccinium myrtillus* (BILBERRY JUICE, Sottobosco Paoli, Civezzano, Italy), 200 ml/day; indication not reported	Warfarin 27.5 mg/w (since 1.5 year)	Reduction of anticoagulant therapy efficacy INR 1.6; 4 days; Possible [4]	Herbal drug withdrawn, warfarin dosage increase (32.5 mg/w); completely recovered

F, 63	*Spirea ulmaria, Taraxacum officinale, Sambucus nigra, Betula alba, Urtica dioica, Cynara scolymus, Amarena, Ttriticum repens, Tilia cordata* (AFFILINE, Nahrin AG, Saren, Switzerland), 1 spoon/day and *Spirulina maxima*, *Urtica Dioica*, Papaya extract (LINPHACELL Nahrin AG, Saren, Switzerland), 4 tablets/day; weight loss diet	Acenocoumarol 20 mg/w (since 2 years)	Reduction of anticoagulant therapy efficacy INR 1.37; 1 month; Possible [2]	Herbal drugs withdrawn, Acenocoumarol dosage increase (22 mg/w); improvement

M, 79	vitamins (C,E), fish oil, lutein, zeaxantin, minerals (PRESERVISION 3, Bausch & Lomb incorporated Rochester, NY), 1 capsule/day; macular degeneration	Acenocoumarol 11 mg/w (since 15 years)	Reduction of anticoagulant therapy efficacy INR 1.50; 6 days; Possible [3]	Supplement withdrawn, Acenocoumarol dosage increase (12 mg/w); improvement

**Table 2:** Reports of INR increase associated with use of natural remedies.

Sex, age (years)	Product, dosage, and indication	Concomitant medications and notes	Adverse drug reaction, time to onset, and Naranjo score	Action taken and outcome
M, 77	*Arnica compositum* (ARNICA CREAM HEEL, Heel Inc., Albuquerque, NM), dosage not reported; myalgia	Warfarin 35 mg/w	Overanticoagulation INR 10.6; 1 month; Possible [4]	Konakion administration; herbal drug withdrawn; completely recovered

F, 42	*Arnica compositum; *calendula; *Hamamelis; Millefolium; *belladonna*; Aconitum; *mercurius solubilis hanemanni*; *hepar sulfuris*;Bellis perennis; Echinacea; Hypericum *(ARNICA CPR HEEL, Heel Inc., Albuquerque, NM), 2 tablets/day; myalgia	Warfarin	Overanticoagulation; Possible [4]	Unknown

F, 73	*Boswellia serrata *dry extract (D E) 95%, 1500 mg/day; osteoarthritis	Warfarin	Overanticoagulation increase of INR; 10 days; Probable [6]	Herbal drug withdrawn; completely recovered

F, 64	*Boswellia serrata* DE 95%, 1200 mg/day; osteoarthritis	Warfarin	Overanticoagulation increase of INR; 10 days; Probable [6]	Herbal drug withdrawn; completely recovered

M, 61	*Passiflora spp, Foenicum vulgare, Melissa officinalis,Glycyrrhiza glabra* (COLISOLVE, Farmacia Legnani, Milano, Italy), dosage not reported; *Pimpinella anisum, Glycyrrhiza glabra, Forniculum vulgare, Ocimum basilicum, Matricaria recutita* (VITABIOSA, Biosa Danmark, Frederiksværk, Denmark), dosage not reported; *Boswellia serrata *DE, *Ficus carica *mother tincture; horse colostrum; *Mucosa compositum*, dosage not reported; indication not reported	Warfarin (since 2 years)	Overanticoagulation INR 8; Possible [4]	Unknown
